# Conditions influencing the appearance of thermal windows and the distribution of surface temperature in hauled-out southern elephant seals

**DOI:** 10.1093/conphys/coaa141

**Published:** 2021-01-23

**Authors:** Alicia I Guerrero, Tracey L Rogers, Maritza Sepúlveda

**Affiliations:** 1 Centro de Investigación y Gestión de Recursos Naturales (CIGREN), Instituto de Biología, Facultad de Ciencias, Universidad de Valparaíso, Gran Bretaña 1111, Playa Ancha, Valparaíso 2360102, Chile; 2 Evolution and Ecology Research Centre, School of Biological, Earth and Environmental Sciences, University of New South Wales, High St., Sydney 2052, Australia; 3 Núcleo Milenio INVASAL, Concepción 4030000, Chile

**Keywords:** Heat dissipation, marine mammal, moult, pinniped, thermography, thermoregulation

## Abstract

Pinnipeds (true seals, sea lions and walruses) inhabit two thermally different environments, air and water, so need to make continuous adjustments to maintain a balanced body temperature. The thermal isolation properties of thick blubber keep warmth within the body’s core, ideal for mammals while in the water; however, when on land, this thick blubber makes it difficult to lose heat. Some pinnipeds use thermal windows, discrete patches where temperature changes on their body surface, as a mechanism to dissipate excessive heat. We identify the factors that correlate with the appearance of thermal windows and changes in body surface temperature on southern elephant seals, *Mirounga leonina*, while they are hauled out ashore. Infrared thermography was used to measure surface temperature of the seals. Temperature was lower on the torso than the flippers and head, suggesting that not all body sites have the same role in thermal balance. Air temperature was the main driver of variation in the surface temperature of the seals’ flippers and head; seals cool their superficial tissues when the air temperature is below ~ 2°C. This minimizes heat loss by reducing the thermal gradient between their skin and the ambient air. Wind speed was the main predictor of whether thermal windows appear on a seals’ body surface. When wind speed was minimal, thermal windows occurred more often, which may be associated with either hair and skin drying, or producing thermal conditions for hair and skin regrowth. The type of aggregation (huddled or alone) influenced the surface temperature of the fore flippers; however, we did not find statistical influence of the seal’s sex, state of moult, or the substrate on which they were hauled out (kelp or sand). Understanding how animals maintain their thermal balance is important if we are to predict how they will respond to future climate change.

## Teaser

Using thermographic images, we found that southern elephant seals use their flippers as body heat controllers, regulating their temperature in response to ambient conditions, especially air temperature. The appearance of thermal windows, specific areas on the body showing higher temperatures, occurred preferentially when there was no wind.

## Introduction

The mammalian thermoregulatory strategy allows mammals to occupy a range of aquatic environments, including shallow and deep waters, as well as tropical and polar regions. However, the mechanisms that mammals use to maintain thermal balance in the aquatic environment are not necessarily the same as those used by mammals on land. For pinnipeds (true seals, sea lions and walruses), maintaining a balanced core body temperature requires continuous thermal adjustments. These amphibious endotherms must adapt to drastically different thermal demands when moving from the aquatic to the terrestrial environment. Water has a thermal conductivity nearly 25 times greater than air (Bonner, 1984; Nienaber *et al.*, 2010), which implies that heat loss rates are substantially higher when animals are in the water compared with when they are on land (Mauck *et al.*, 2003). An amphibious lifestyle, therefore, requires an efficient thermoregulatory system that prevents excessive heat loss when in the water without causing excessive heat retention when in air (Gentry, 1973).

Having a large body with reduced surface-to-volume ratios (Innes *et al.*, 1990) is one of the mechanisms that minimizes heat loss in aquatic mammals. This decreases the relative area across which heat transfers to the aquatic environment (Pabst *et al.*, 1999). Beside this, aquatic mammals have streamlined and compact bodies with few protruding appendages, which prevents rapid heat dissipation (Riedman, 1990). Their thick blubber is an effective insulating layer that reduces thermal conductance and therefore heat loss to the surrounding environment (Scholander *et al.*, 1950; Bagge *et al.*, 2012; Liwanag *et al.*, 2012). While on land heat retention is an issue, here pinnipeds are thought to avoid overheating via behavioural responses such as entering the water to cool (Tarasoff and Fisher, 1970), moving onto wet sand (Whittow, 1978) or adjusting their posture to expose their flippers (Beentjes, 2006).

Flippers are proposed to be important in heat dissipation (Irving and Hart, 1957; Hart *et al.*, 1959; Kvadsheim and Folkow, 1997) since they are not well insulated, lack thick blubber and dense fur and consequently have more variable surface temperature compared to the body (e.g. harbour seals, *Phoca vitulina*, Hart *et al.*, 1959). However, few pinniped species have thermal windows, small areas on the body’s surface (e.g. trunk, neck) that function as temporary heat dissipaters (Mauck *et al.*, 2003). Thermal windows have visible borders clearly showing higher temperatures than the surrounding area (Mauck *et al.*, 2003; Erdsack *et al.*, 2012). This phenomenon would allow pinnipeds to rid excess heat while they are on land. Thermal windows have been reported in the harbour seal, harp seal *Pagophilus groenlandicus*, grey seal *Halichoerus grypus* (Mauck *et al.*, 2003) and walrus *Odobenus rosmarus* (Rodríguez-Prieto *et al.*, 2013) but do not seem ubiquitous in all pinnipeds. In Weddell seals *Leptonychotes weddellii*, for example, temperature does not vary over the seals body (Mellish *et al.*, 2015). Thus, although thermal windows are thought to be a thermoregulatory strategy to release excess heat, the mechanisms driving their presence are not fully understood.

The use of infrared thermography allows continuous measurements of body surface temperature without the need of physical contact with the test subject. This is particularly useful to study large wild animals in remote locations where it is not possible to conduct trials with a large number of temperature sensors (Cena and Clark, 1973; McCafferty, 2007). Infrared thermography has proven useful in thermal studies of insects, reptiles, birds and mammals (McCafferty *et al.*, 1998; McCafferty, 2007). In pinnipeds in particular, infrared thermography has been used to study captive animals, including the Steller sea lion *Eumetopias jubatus* (Nienaber *et al.*, 2010) and grey, harp and harbour seals (Øritsland, 1968; Mauck *et al.*, 2003; Nienaber *et al.*, 2010; Paterson *et al.*, 2012). These captive pinniped studies focused on understanding how surface temperature patterns vary by season, during exercise, or after animals exit the water. Thermographic technology studies on wild pinnipeds include the Weddell seal (Mellish *et al.*, 2015), northern elephant seal *Mirounga angustirostris* (Norris *et al.*, 2010), grey seal (McCafferty *et al.*, 2005), harbour seal (Erdsack *et al.*, 2012) and southern elephant seal *Mirounga leonina* (Chaise *et al.*, 2019). Studies of pinnipeds in their natural environment have focused on understanding thermoregulatory patterns associated with hauling out voluntarily or in training situations, lactation, moulting and huddling behaviour.

The southern elephant seal is an interesting model to examine thermoregulation because they are a large amphibious mammal that lives across a wide thermal range in both terrestrial and aquatic habitats. Indeed, the southern elephant seal is the largest of the pinnipeds, where males can weigh up to 3800 kg (Ling and Bryden, 1981; Hindell and Perrin, 2009) so that heat dissipation while hauled out ashore is crucial. They exhibit the most extreme sexual dimorphism of any mammal, with males up to nine times larger than the females (Hindell and Perrin, 2009) so that there may be intersexual differences in thermoregulatory strategies. Southern elephant seals have a nearly circumpolar distribution in the Southern Ocean (Le Boeuf and Laws, 1994), and although currently most of the global population inhabits sub-Antarctic waters, some populations haul out ashore for many weeks at a time in extremely cold high-latitude sites on the Antarctic continent (Ling and Bryden, 1992), while others use warm temperate sites, such as the resident population in Peninsula Valdes (42° 31′ S), Argentina (Campagna and Lewis, 1992), as well as the frequent sightings further north (e.g. 29° 02′ S) (Cárcamo *et al.*, 2019). Furthermore, historically, there were large colonies on the western Bass Strait islands and northwest coast of Tasmania, Australia (Ling and Bryden, 1992; Bryden *et al.*, 1999; Ling, 1999) and in Juan Fernandez Islands, Chile (Cárcamo *et al.*, 2019). If we are to predict how animals respond to future changes in climatic conditions, we need to understand how they maintain their thermal balance; this is especially important as episodes of extreme warming are becoming stronger and more frequent (Russo *et al.*, 2014). Here, we evaluate how different factors (e.g. sex, social behaviour, the substrate they haul out upon, environmental conditions) influence one of the main avenues for body heat control in the southern elephant seal: the distribution of surface temperature. We hypothesize that the appendages are the main regulators of body temperature in southern elephant seals and will, therefore, actively respond to changes in environmental conditions. To do this, we use infrared thermography to measure the surface body temperature of wild southern elephant seals under varying behavioural and environmental conditions.

## Methodology

### Data collection

Southern elephant seals were sampled while hauled out ashore during their annual moult, at two colonies at different latitudes, one a sub-Antarctic and the other an Antarctic site, in order to capture a range of weather conditions. This included 52 southern elephant seals on Elephant Beach (62° 12′ 01′′ S, 58° 59′ 56′′ W), King George Island, Antarctica, during February 2019; and 11 southern elephant seals on Jackson Bay (54° 27′ 00′′ S, 68° 57′ 53′′ W), Tierra del Fuego Island, Chile, during March 2019.

**Figure 1:**
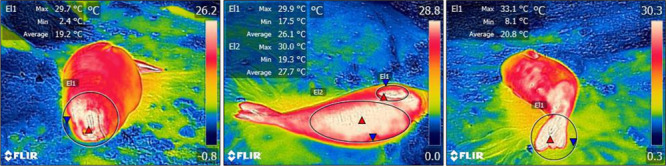
Thermal measurements per body region using FLIR Tools software (Maximum surface temperature is indicated by the red triangles).

For each seal, thermograms were obtained using a FLIR E6 camera (FLIR Systems, Danderyd, Sweden), with an infrared resolution of 160 × 120 pixels, MSX technology (320 × 240 pixels) and thermal sensitivity < 0,06 at 30°C. The camera was calibrated by FLIR prior to the study. Hauled-out seals were approached by foot. At least three thermograms were taken at 1–3 m from each seal, such that the subject filled the frame, including the frontal (head), rear (hind flippers) and lateral side (either left or right, or both) of the seal (Fig. 1). Images were obtained, when possible, at a 90 degree angle from the target, in order to minimize the influence of curved surfaces (Ash *et al.*, 1987).

Environmental data, air temperature, wind speed and relative humidity were collected alongside the test subject, using a hand-held Digital Anenometer PM6252B (PEAKMETER, China). The instrument accuracy was ±1.5°C for temperature, ±2% for wind speed and ±3% for relativehumidity.

The behavioural data collected included whether the test subject was alone (isolated) or alongside other seals (in a huddle), the substrate type on which they were hauled out (kelp or sand) and their moult stage. Moult stage was classified as pre-moult, when shedding of old hair/skin had not started; mid-moult, when areas of both new and old hair/skin were clearly visible; and post-moult, when new hair covered the entire body surface. No individuals were in the initial moult stage (i.e. when skin shedding was minimal; Chaise *et al.*, 2019); therefore, this stage was not included.

### Image analysis

Thermograms were analysed using FLIR Tools, version 5.13 (FLIR Systems ^©^2016) in a rainbow colour scheme. FLIR Tools was used to adjust each image for ambient temperature, relative humidity and distance from the seal. Emissivity was set at 0.98 for each image within the software, value reported for pelage (Hammel, 1956; Norris *et al.*, 2010; Mellish *et al.*, 2015). Using the ellipse tool within the software, each image was scored for the maximum surface temperature of the head, fore flippers, hind flippers and torso of each animal, as shown in Fig. 1. As having a wet coat obscured the reading of temperature variation in the underlying body surface, we used only test subjects that had a dry pelage for statistical analysis. Only two female elephant seals were completely wet and were therefore excluded from statistical analyses. When thermal windows were identified in a seal, we registered their number and maximum surface temperature.

### Data analysis

To determine whether body regions differed in temperature, we conducted an analysis of covariance (ANCOVA), with air temperature as the covariant, body region (head, torso, fore and hind flippers) as the independent variable and surface temperature as the response variable.

To evaluate whether there were differences in surface temperature between males and females, we conducted an ANCOVA where the surface temperature of each body region was the response variable, sex was the explanatory variable and air temperature was the covariant. In the same manner, we tested for differences between elephant seals hauling out in huddles or isolated, between different stages of the moult and between types of surface (kelp vs sand) used for hauling out.

We evaluated how the surface temperature of each body region varied along different ranges of environmental traits using generalized additive models (GAMs) in the mgcv package in R (Wood, 2017). These models included the predictor variables air temperature, relative humidity and wind speed, and the response variable was the surface temperature of each body site in separate models. Models were ranked via Akaike’s information criterion with a correction for small sample size (AICc) (Burnham and Anderson, 2002; Johnson and Omland, 2004) and the best fit model was selected according to the lowest AICc.

Body heat loss is dependent on the thermal difference between the body surface and air temperature (Kvadsheim and Folkow, 1997). To investigate the effect of air temperature variations on this thermal difference (or thermal gradient), we conducted a GAM analysis, where the thermal gradient between air and each body region (head, torso, fore and hind flippers) was the response variable and air temperature the only predictor.

We conducted general linear models (GLM) to identify which environmental traits promoted the presence of thermal windows. To do this, we used presence/absence of thermal windows as a binomial response variable and air temperature, humidity and wind speed as explanatory variables. The models included different combinations of the explanatory variables and the best model was selected usingAICc.

The level of significance for all statistical tests was set at *P* < 0.05. Analyses were conducted using R Studio (R Core Team, 2013).

## Results

A total of 404 thermograms, corresponding to 45 females and 18 males, were obtained. Torso was the body region with the lowest surface temperature (14.8 ± 7.5°C) with values < 10°C reported in 36% of the seals. In contrast, hind flippers had the highest surface temperature (24.9 ± 4.2°C), followed by head (24.1 ± 5.0°C) and fore flippers (22.7 ± 5.4°C). Surface temperature differed significantly among body regions (ANCOVA, *F* = 49.59, *P* < 0.001); however, only the torso was different to all other body regions (Tukey, *P* < 0.001), whereas fore, hind flippers and head did not differ in surface temperature (Tukey, *P* > 0.050). Although all body sites had higher surface temperature in males compared to females, none of these differences were significant (ANCOVA, head: *F* = 0.08, *P* = 0.775; torso: *F* = 0.03, *P* = 0.854; fore flippers: *F* = 1.84, *P* = 0.182; hind flippers: *F* = 3.31, *P* = 0.075).

**Figure 2:**
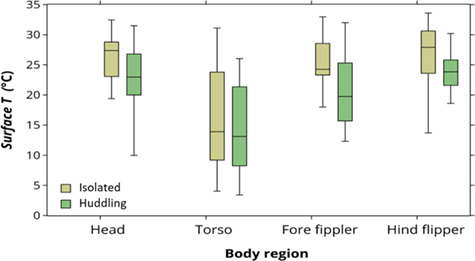
Box and whisker representation of the maximum surface temperature (*T*) per body region according to type of aggregation, for southern elephant seals (*n* = 61).

The type of aggregation of each southern elephant seal (isolated = 21, in huddles = 40) only affected the temperature of fore flippers significantly (ANCOVA, *F_2_* = 6.79, *P* = 0.013; Fig. 2). Although the torso, head and hind flippers had higher temperatures when seals were isolated, these differences were not significant (head: *F_2_* = 1.89, *P* = 0.174; torso: *F_2_* = 0.23, *P* = 0.629; hind flippers: *F_2_* = 3.13, *P* = 0.082).

Surface temperature did not vary significantly according to stage of moulting. The torso had temperatures only marginally different among different moult stages (ANCOVA, *F_2_* = 3.08, *P* = 0.054) with post-moult seals (*n* = 17) exhibiting higher temperatures than those in pre- (*n* = 35) and mid-moult (*n* = 9) stages. Head and flippers temperatures were slightly higher during the mid-moult stage; however, these differences were not significant **(**head: *F_2_* = 1.47, *P* = 0.239; fore flippers: *F_2_* = 1.89, *P* = 0.164; hind flippers: *F_2_* = 0.64, *P* = 0.531).

The type of substrate (kelp = 35 seals, sand = 26 seals) did not influence the surface temperature of their heads (ANCOVA, *F_1_* = 0.37, *P* = 0.543), torsos (*F_1_* = 0.52, *P* = 0.471), fore flippers (*F_1_* = 2.18, *P* = 0.147) or hind flippers (*F_1_* = 3.13, *P* = 0.082).

**Figure 3:**
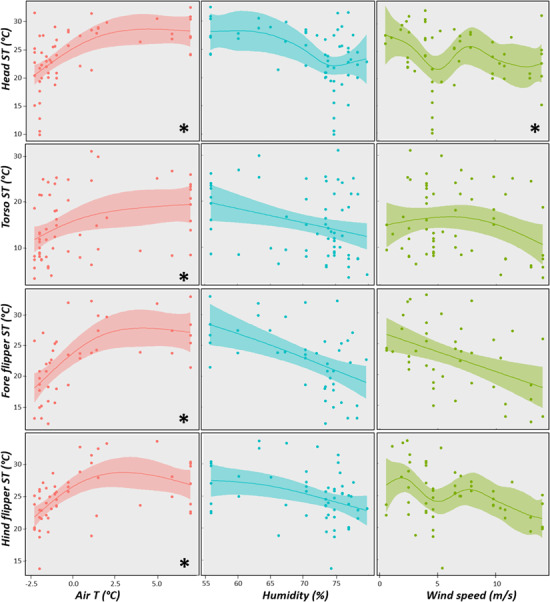
Effect of air temperature (air T), relative humidity and wind speed, on the surface temperature of different body sites (head T, torso T, fore flipper T and hind flipper T) of the southern elephant seal, where asteriks indicate the variables included in the best supported model.

Regarding the effects of environmental traits on the surface temperature of southern elephant seals, the variation in head temperature was best explained by the model including air temperature and wind speed (AICc = 0), although the model including humidity had a very similar AICc value (0.36) and therefore was equally supported (see Table S1 of Supplementary material). The model including air temperature and wind speed explained 43% of the deviance, and the inclusion of humidity added an extra 2%. Head temperature increased with ambient air temperature but it remained constant after air temperature reached ~ 2.5°C. Conversely, head temperature tended to decrease as wind speed increased (GAM, *r^2^* = 0.39; s(air temperature) *P* < 0.001, s(wind speed) *P* = 0.045; Fig. 3, Table S1). The highest supported model for torso temperature was the one that included air temperature and it only explained 16% of the deviance (GAM, *r^2^* = 0.14; s(air temperature) *P* = 0.010; Fig. 3). Similarly, air temperature was the only variable included in the best supported model to explain thermal variations in both fore flippers (GAM, *r^2^* = 0.42; s(air temperature) *P* < 0.001; Fig. 3) and hind flippers (GAM, *r^2^* = 0.33; s(air temperature) *P* < 0.001; Fig. 3) and explained 45% and 36% of the deviance, respectively. Torso and flippers temperature tended to increase as air temperature rose, although this increase ceases when air temperature reaches ~ 2 °C.

**Figure 4:**
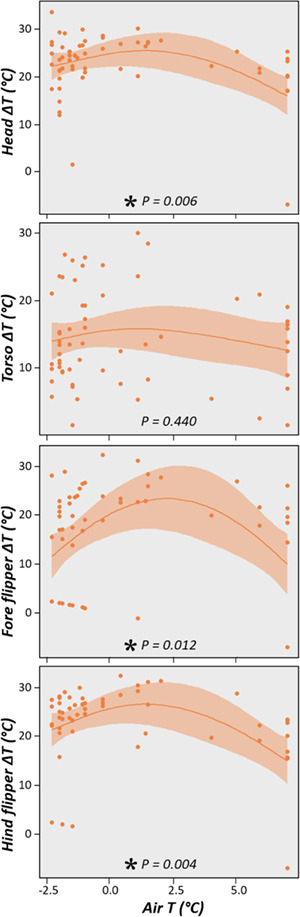
Effect of ambient air temperature (air T) on the temperature difference (ΔT = ambient air temperature − surface temperature) for each body site.

The thermal gradient between air and the animal’s surface tended to increase slightly as air temperature rose; however, when air temperature reached ~ 2°C the temperature difference started to diminish (Fig. 4). This trend was similar for head (GAM, *r^2^* = 0.174; s(air temperature) *P* = 0.006), fore flippers (GAM, *r^2^* = 0.160; s(air temperature) *P* = 0.012) and hind flippers (GAM, *r^2^* = 0.183; s(air temperature) *P* < 0.004); however, the relationship between air temperature and the thermal gradient of the torso was more linear (edf = 1.7) compared to other parts of the body although not significant (GAM, *r^2^* = 0.014; s(air temperature) *P* = 0.440).

**Figure 5:**
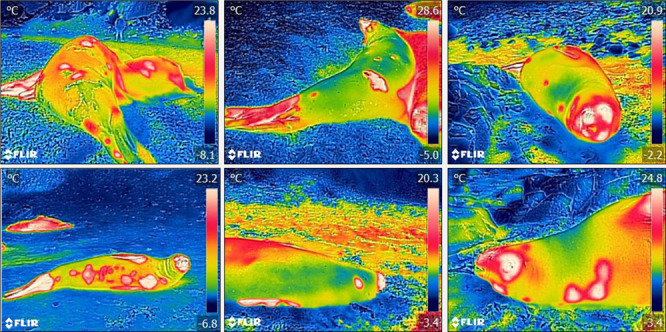
Thermograms of southern elephant seals showing thermal windows in different parts of the body, including torso, belly, neck and hind flippers.

Thermal windows were identified in 24 out of the 63 seals studied. They usually had circular or irregular shapes and were located mainly on the torso but also around the neck, belly and hind flippers (Fig. 5). The number of thermal windows ranged from 1 to 12 per seal and their mean temperature was 20.7 ± 4.4°C. The minimum temperature recorded for a thermal window was 10.1°C whereas the maximum was 28.5°C. The mean temperature difference between a thermal window and the surrounding area was 9.1 ± 4.2°C, with thermal windows having up to 3.5 times higher temperature than the surrounding body area.

**Figure 6:**
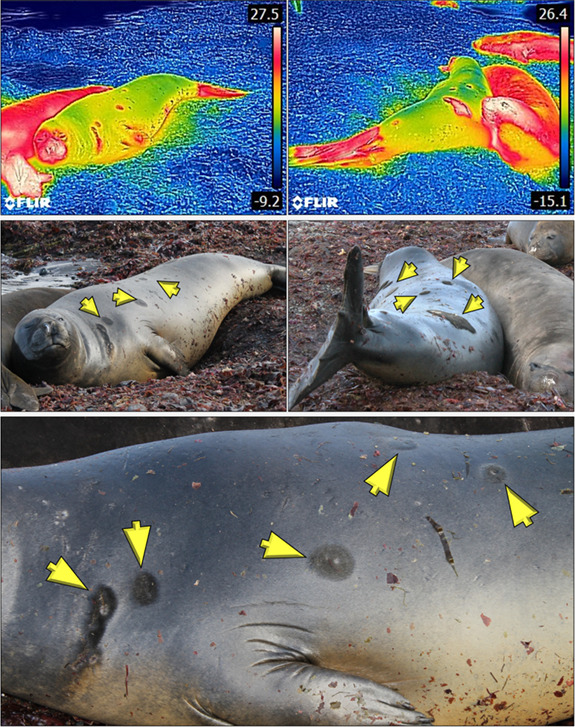
Series of thermograms and digital photographs of a southern elephant seal after exiting the water, showing circular patches of dry fur (yellow arrows) which were identified as thermal windows in the thermograms (digital photographs by Andrea Colilef).

Only two elephant seals were completely wet at the time the photographs were taken, and both had thermal windows. For seals with a mosaic of wet hair with dry patches, the patches of dry hair correspond to the location identified as thermal windows in the thermograms, these thermal patches were visible with the naked eye (Fig. 6). However, when the seals had completely dry hair, yet thermal windows were identified in the thermograms, thermal windows were invisible to the naked eye.

**Figure 7:**
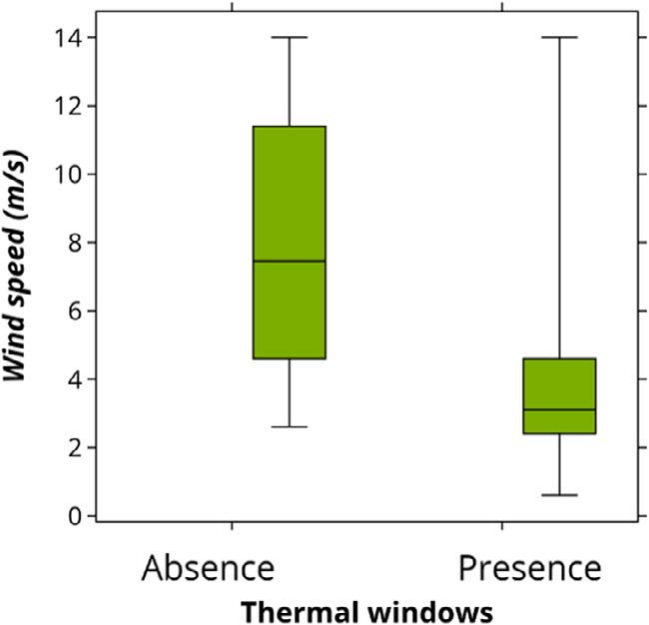
Box and whisker representation of the effect of wind speed on the occurrence of thermal windows in southern elephant seals.

Although the model that best explains the presence of thermal windows includes wind speed only (ΔAICc = 0, Table S2), the two alternate models that include alongside wind speed, humidity as well as temperature, also had high support (within 2 ΔAICc units, Table S2). Thermal windows are more likely to occur on seals at low wind speeds (GLM, *P* < 0.001; Fig. 7), when humidity was low and temperature was high.

## Discussion

One of the main advantages of infrared technology is its ability to relate changes in surface temperature to particular physiological states or behaviours (McCafferty, 2007), which may occur as responses to environmental changes. We analysed surface temperature patterns in the southern elephant seal in order to determine how they fluctuate under a range of environmental factors. Our study found that southern elephant seals, like some other pinnipeds, develop thermal windows and allowed us to infer the environmental conditions that promote their appearance. We found differences in surface temperatures among body regions of southern elephant seals, which suggests that not all body sites have the same role in controlling body heat flow.

The appendages and head had higher surface temperatures than the torso, and this was driven mainly by air temperature. Since surface temperature is linked to underlying blood flow (Gentry, 1973), this suggests that southern elephant seals constantly adjust the peripheral blood flow in head and flippers in response to environmental conditions, in particular air temperature. Similarly, other species of pinnipeds such as Steller’s sea lions and harbour seals (Nienaber *et al.*, 2010) display different surface temperatures depending on body site. Conversely, the Weddell seal has uniform surface temperatures throughout its body during the Antarctic summer (Mellish *et al.*, 2015). In pinnipeds, flippers are thought to play an important role in controlling heat dissipation, as their temperature usually correlates with air temperature (Cuyler *et al.*, 1992; Choi *et al.*, 1997). These appendages, usually with bare or sparsely haired surfaces, can control heat loss via regulation of peripheral blood flow (Mauck *et al.*, 2003). In harp seals, fore and hind flippers increase their surface temperature in up to 22°C after exercise, which supports the idea that pinnipeds regulate heat loss mainly by means of controlled heat dissipation from the appendages (Øritsland, 1968). Our results suggest that flippers actively respond to changes in ambient conditions. Although the surface temperature of the head was also associated with variations in the surrounding conditions, some sections of the head, such as eyes and muzzle, may not be considered heat regulators since they are poorly insulated and need to maintain a high level of irrigation in order to operate (Dehnhardt *et al.*, 1998; Nienaber *et al.*, 2010).

Regarding the torso temperature of southern elephant seals, where air temperature only explains a small portion of the deviance (16%), there must be other factors, potentially endogenous, driving their surface temperature. The torso temperature varied greatly, from values that almost equalled those of air, to temperatures up to 30°C higher; however, this thermal difference between air and body surface was not driven by air temperature (Fig. 4). This independence of the torso temperature with its surrounding environment may be due to the role of blubber as insulator. Where animals do not use fur as their primary insulator, which is the case of southern elephant seals as the most hairless phocid (Gentry, 1973), blubber is expected to play a more important role in the control of core body heat (Guerrero and Rogers, 2019). The torso, unlike the appendages, has a thick layer of blubber that provides insulation from ambient conditions and is therefore considered of minor importance in heat loss regulation (Øritsland, 1968). This potentially explains why air temperature was not an important driver of the surface temperature of the torso. However, the level of insulation provided by the blubber layer can be adjusted via constriction or dilation of the blood vessels trespassing it. (Molyneux and Bryden, 1978; Guerrero and Rogers, 2019). Thus, depending on the thermal needs of each animal, their torso may exhibit very different surface temperatures when heat retention or dissipation occurs.

The great variation found in surface temperatures of the torso could also be associated with the moulting process. The moult is an energetically expensive process, where other physiological processes, such as thermoregulation, can be affected (Paterson *et al.*, 2012). During the moult, seals need to allow blood circulation for new hair growth (Worthy, 2001). The phocid epidermis requires high temperatures for optimal renewal (Chaise *et al.*, 2019), which could imply high rates of heat loss for southern elephant seals living in a cold environment. Additionally, elephant seals fast during the moult; therefore, they need to use up their blubber reserves, which leads to thinning of blubber and consequently to a reduction of its insulating properties (Iverson, 2009). Since during the study southern elephant seals were in different stages of the moult, the variation in surface temperatures observed could be driven by a combination of intrinsic factors (e.g. moulting, heat retention or dissipation) and to a lesser extent, extrinsic factors (e.g. ambient conditions).

Chaise *et al.* (2019) found that southern elephant seals aggregated more during the mid-stage of moult and during colder days, which may help them cope with the thermoregulatory costs of both moult and weather conditions. Although we did not survey the area to record the aggregation behaviour of all seals, we did photograph a larger number of seals huddling when air temperature was below 1°C (33 vs 7 at higher temperatures), which also explains why we observed higher surface temperatures in isolated seals as compared to aggregated seals. Other pinnipeds, such as the California sea lion, *Zalophus californianus*, also benefit from this huddling behaviour when in cold weather (Liwanag *et al.*, 2014). Huddling is a social thermoregulatory strategy that allows animals to save energy in maintaining thermal balance and thus reallocate this energy to other functions (Gilbert *et al.*, 2010). Considering that elephant seals fast during the moult and therefore energy is a limiting factor, this behaviour presents important thermoregulatory advantages.

Heat loss is mainly driven by the temperature difference between an animal’s surface and the surrounding (Kvadsheim and Folkow, 1997; Walcott *et al.*, 2020). The surface temperature of hauled-out southern elephant seals was highly variable, similar to another Antarctic mammal, the Weddell seal (Mellish *et al.*, 2015). In our study, the thermal gradient pattern was similar for flippers and head (Fig. 4): at low air temperatures (~ −2.5°C) the thermal gradient was small but it increased until air temperature reached ~ 2°C and then it decreased again. Figure 3 shows that surface temperature decreases with air temperatures lower than ~ 2°C, but the animal’s surface temperature diminished at a higher rate than air, reducing the thermal gradient. This suggests that when air temperature falls below 2°C animals reduce the thermal gradient in order to reduce heat loss. We could not include measurements at lower air temperatures but, potentially, the thermal gradient is even smaller. There is increasing evidence that high-latitude species may minimize this thermal gradient between body surface and the cold surrounding air (Mellish *et al.*, 2015) via regulation of dermal perfusion with arteriovenous anastomoses (Krmpotic *et al.*, 2018; Walcott *et al.*, 2020).

For each body region, surface temperature exhibited a steady increase until air temperature reached ~ 2°C after which surface temperature of southern elephant seals reached a plateau (Figure 3). These data can provide insights into the critical temperatures of the species (Mellish *et al.*, 2015). Potentially, at air temperatures lower than 2°C southern elephant seals have to make physiological adjustments in order to conserve heat, such as lowering the temperature of superficial tissues and reducing peripheral blood flow. Cooling superficial tissues in order to avoid heat loss during cold weather could compromise the completion of the moulting process, which requires higher surface temperatures. These animals, therefore, need to make continuous adjustments in order to balance thermoregulation and moulting during this period. A study of Weddell seals showed that their surface temperature reaches a plateau when environmental temperatures are above −15°C (Molyneux and Bryden, 1978), much lower than southern elephant seals. Weddell seals are the southernmost living mammal (Mellish *et al.*, 2015); therefore, they operate at a lower temperature range than our study species, but these data suggest that for southern elephant seals extremely low environmental temperatures may be energetically costly. This might imply that southern elephant seals will be negatively impacted or forced to change their distribution range, during events of extreme-cold weather, since the moulting process in these conditions would be more challenging and, therefore, the completion of their life cycle could become compromised.

Early observations on the skin temperature of southern elephant seals described warm circular-shaped areas in irregular patterns on the trunk (Krumbiegel, 1933). Our study further reinforces the idea that these warmer areas are thermal windows, although their function is still unclear. The identification of body parts with relatively high temperature can be associated to an animal’s anatomy and physiology (McCafferty, 2007). The presence of arteriovenous anastomoses in the skin of phocid species is important in thermoregulation, in particular when animals are on land in order to increase the dissipation of body heat (Molyneux and Bryden, 1978; Erdsack *et al.*, 2012). Could arteriovenous anastomoses be denser in areas where thermal windows appeared? Molyneux and Bryden (1978) found that the mean density of arteriovenous anastomoses in southern elephant seals was homogenous over the entire body surface, which suggests that thermal windows are not necessarily associated with areas of denser vascularization but rather with adjustments of blood flow beneath those areas.

Øritsland (1968) found that when harp seals were subjected to exercise, in several cases warm spots appeared in irregular patterns on their torso surface, which the author described as a mechanism to increase heat dissipation and thus regulate their temperature. Mauck *et al.* (2003) found that the initial size and growth of thermal windows in harbour seals were dependent on ambient temperature and season, where thermal windows appeared faster and grew wider in the summer season, when ambient temperatures were higher. Although in the literature thermal windows are mainly associated with the need of losing excess heat, it seems unlikely that this could be the case for southern elephant seals. The study area presents lower temperatures than other sub-Antarctic regions where elephant seals inhabit; therefore, it is more likely that these seals need to conserve heat rather than dissipate it. Additionally, during the moulting period, elephant seals rest most of the time; therefore, they should not need to release heat due to exercise. We hypothesize that thermal windows could be an efficient strategy for hair and skin drying. Mauck *et al.* (2003) found thermal windows in harp, harbour and grey seals in captivity, when they exited the water and therefore had wet hair when this occurred. This produced a ‘forced evaporation’ of water contained in the hairs and, consequently, these areas dried much faster than the rest of the body surface. Our results showed that thermal windows appear in elephant seals that had wet and dry hair. When thermal windows occurred in seals with wet hair, evaporation made these areas dry faster; therefore, spots of dry hair were clearly visible to the naked eye (Fig. 6). This feature could help animals dry their hair in a more efficient manner in cold environments, where heat concentrates in a small area and expands as hair dries. The alternative mechanism could be to release heat homogeneously through the pelage, which would result in relatively low heat dissipation per unit area, and water evaporation would occur at a slower pace (Mauck *et al.*, 2003).

Interestingly, we found that thermal windows also occurred when seals had completely dry hair. This suggests that this mechanism may not only allow an efficient evaporation but it is also used with other purposes. We hypothesize that thermal windows could benefit moulting process by increasing the skin temperature in specific areas and thus promote hair and epidermis regrowth. Because the increase of blood flow to the skin surface under cold weather conditions compromises heat balance, thermal windows should occur when the risk of heat loss is minimal. We found that thermal windows occurred more often with low wind speed. Wind impacts the insulation provided by the pelage. Even for animals with sparse hairs such as southern elephant seals, heat loss is minimal with still air since the pelage maintains a layer of air that serves as an insulating layer. However, the air trapped in the hairs is quickly removed by a slight wind, in particular if animals have low hair density (Tregear, 1965) or lack underfurs, which is the case of southern elephant seals (Krmpotic *et al.*, 2018). Thus, windy environments lead to large convective heat losses (McCafferty *et al.*, 2013). If the purpose of thermal windows is to favour moulting process, therefore, they should appear preferentially when it is less costly (e.g. when there is no wind), in order to avoid excessive heat loss.

It is important to note, however, that there are other environmental factors not considered in the model such as solar radiation that could potentially be associated with the occurrence of thermal windows in this species. Another limitation to this study is the relatively narrow range of environmental traits analysed. An *in situ* assessment that considers a wider spectre of environmental conditions could provide more information on the thermal thresholds of these animals. As climate keeps changing, it is of key importance to understand the mechanisms of heat regulation in mammals, in particular those inhabiting extreme environments, in order to infer how these animals will adapt to events of extreme weather.

In conclusion, our results suggest that the main thermal regulators of southern elephant seals are their fore and hind flippers, as they respond more actively to changes in environmental conditions. Our finding of thermal windows (most of them in the torso) suggest that elephant seals use this mechanism for an efficient hair drying and/or to favour moulting process by providing adequate surface temperatures for hair and skin regrowth.

## Supplementary Material

Supplementary_material_coaa141
