# Supplementary material for: Conditions influencing the appearance of thermal windows and the distribution of surface temperature in hauled-out southern elephant seals
Source: Conserv Physiol. 2021 Jan 23;9(1):coaa141. doi: 10.1093/conphys/coaa141 (PMC8216107; doi:10.1093/conphys/coaa141)
Supplement: Supplementary_material_coaa141 [file supplementary_material_coaa141.docx]

**Supplementary material**

**Table S1.** Generalised additive model selection of variations in surface temperature (T) in southern elephant seals. The values for each candidate model, where ‘s’ indicates a smooth function, include degrees of freedom (d.f.), log-likelihood, AICc value and difference between the AICc value of each candidate model and the best fit model (ΔAICc). The highest level of support is indicated by the lowest ΔAICc value. The table only includes the models within 2 ΔAICc units and the null models.

| **Models** | **d.f** | **Log-likelihood** | **AICc** | **ΔAICc** |
| --- | --- | --- | --- | --- |
|  |  |  |  |  |
| ***Head T response*** |  |  |  |  |
| s(air temperature + wind) | 5.9 | -159.8 | 333.2 | 0.0 |
| s(air temperature + humidity + wind) | 7.0 | -158.6 | 333.6 | 0.4 |
| s(air temperature + humidity) | 5.8 | -161.0 | 335.0 | 1.8 |
| Null | 2.0 | -176.0 | 356.3 | 23.1 |
|  |  |  |  |  |
| ***Torso T response*** |  |  |  |  |
| s(air temperature) | 4.1 | -199.3 | 407.6 | 0.0 |
| s(air temperature + wind) | 6.8 | -196.5 | 408.6 | 1.0 |
| s(air temperature + humidity) | 5.1 | -199.0 | 409.3 | 1.7 |
| s(humidity) | 3.0 | -201.5 | 409.5 | 1.9 |
| Null | 2.0 | -204.6 | 413.4 | 5.8 |
|  |  |  |  |  |
| ***Fore flippers T response*** |  |  |  |  |
| s(air temperature) | 4.8 | -118.0 | 247.3 | 0.0 |
| Null | 2.0 | -130.6 | 265.6 | 18.3 |
|  |  |  |  |  |
| ***Hind flippers T response*** |  |  |  |  |
| s(air temperature) | 4.9 | -143.5 | 298.2 | 0.0 |
| s(air temperature + humidity) | 5.9 | -142.8 | 299.2 | 1.0 |
| Null | 2.0 | -155.8 | 315.9 | 17.7 |

**Table S2.** Explanatory models to examine the influence of environmental variables on the presence of thermal windows in southern elephant seals. The values for each candidate model include log-likelihood, AICc value and difference between the AICc value of each candidate model and the best fit model (ΔAICc).

| **Model** | **Log-likelihood** | **AICc** | **ΔAICc** |
| --- | --- | --- | --- |
| Wind speed | -31.2 | 66.6 | 0 |
| Air temperature + wind speed | -31.0 | 68.4 | 1.8 |
| Humidity + wind speed | -31.1 | 68.5 | 1.9 |
| Air temperature + humidity + wind speed | -30.9 | 70.5 | 3.9 |
| Null | -41.8 | 85.8 | 19.2 |
| Humidity | -40.9 | 86.0 | 19.4 |
| Air temperature | -40.9 | 86.1 | 19.5 |
| Air temperature + humidity | -40.9 | 88.2 | 21.6 |
